# Remote Control Effect of Li^+^, Na^+^, K^+^ Ions on the Super Energy Transfer Process in ZnMoO_4_:Eu^3+^, Bi^3+^ Phosphors

**DOI:** 10.1038/srep27657

**Published:** 2016-06-09

**Authors:** Weiguang Ran, Lili Wang, Lingling Tan, Dan Qu, Jinsheng Shi

**Affiliations:** 1 Qingdao Agricultural University, Qingdao 266109, People’s Republic of China

## Abstract

Luminescent properties are affected by lattice environment of luminescence centers. The lattice environment of emission centers can be effectively changed due to the diversity of lattice environment in multiple site structure. But how precisely control the doped ions enter into different sites is still very difficult. Here we proposed an example to demonstrate how to control the doped ions into the target site for the first time. Alkali metal ions doped ZnMoO_4_:Bi^3+^, Eu^3+^ phosphors were prepared by the conventional high temperature solid state reaction method. The influence of alkali metal ions as charge compensators and remote control devices were respectively observed. Li^+^ and K^+^ ions occupy the Zn(2) sites, which impede Eu and Bi enter the adjacent Zn(2) sites. However, Na^+^ ions lie in Zn(1) sites, which greatly promoted the Bi and Eu into the adjacent Zn(2) sites. The Bi^3+^ and Eu^3+^ ions which lie in the immediate vicinity Zn(2) sites set off intense exchange interaction due to their short relative distance. This mechanism provides a mode how to use remote control device to enhance the energy transfer efficiency which expected to be used to design efficient luminescent materials.

Phosphor-converted white light emitting diodes (pcWLEDs) are treated as next generation lighting source^1^,^2^. Currently, the most common pcWLEDs employ blue InGaN LED coated with yellow Y_3_Al_5_O_12_:Ce^3+^ (YAG:Ce^3+^) phosphor^3^,^4^. It is un-optimized for indoor use due to emission spectrum deficient in the red spectral region. To enhance red emission and raise color rendering index, a blend of YAG:Ce^3+^ and red emitting phosphor is generally utilized^5^. However, the current commercial red phosphor like Y_2_O_2_S:Eu^3+^ present chemical instability and low absorption in near ultraviolet (UV) region^6^. Hence high efficient and stable red phosphors that can be excited in near UV region should be developed.

The luminescence property of phosphor is known to be strongly affected by crystal lattice environment of the host. At present, adjusting the lattice environment of activators is a hotspot for solid state lighting^7^. In order to improve the luminescence efficiency, site occupation of activators in host lattice has been investigated from different perspectives^5^,^7^,^8^,^9^. Peng^5^ found the site occupancy preference of Mn^4+^ in Sr_4_Al_14_O_25_ is at the Al(4) and Al(5) higher covalent sites rather than the Al(6) site. They believe the high fluorescence intensity and thermal stability are due to the special environment of Mn^4+^ centers. Wang^9^ has improved the thermal stability of CaAlSiN_3_:Eu^2+^ phosphors through neighboring-cation substitution. Tsai^7^ has enhanced the luminescent behavior of Eu^2+^ doped CaAlSiN_3_ phosphors through adjusting the lattice environment of Eu^2+^ ions. And the lattice environment of Eu^2+^ doped CaAlSiN_3_ phosphor has been modified through cation substitution to induce charge variation and a rearrangement of neighboring nitride clusters.

Bi^3+^ is a common activator and there are lots of reports on Eu^3+^ red luminescence enhancement via energy transfer from Bi^3+^ to Eu^3+^ in a variety of hosts^10^,^11^. A free Bi^3+^ ion has 6s^2^ electronic configuration and the absorption band of phosphors doped with Bi^3+^ might be extended to near UV region due to the influence of the host lattice on the outermost electrons^12^,^13^. Lili Wang^14^ has summarized and established the relationships between the positions of energy levels of Bi^3+^ and environmental factor *h*_*e*_ of host materials in dozens of compounds. Recently, Bi^3+^ and Eu^3+^ co-doped ZnMoO_4_ phosphors have been synthesized through solid state reaction and their properties have been discussed^12^. It has been found that there exists a super energy transfer process from Bi^3+^ to Eu^3+^ due to the special S-shaped cluster in ZnMoO_4_. There are three kinds of Zn sites in ZnMoO_4_ and every six nearest Zn atoms form an S-shaped cluster. According to the quantitative relationship between energy levels of Bi^3+^ and host lattice environment established before^14^, the positions of ^1^S_0_→^3^P_1_ transition of Bi^3+^ in three different Zn sites were predicted and then it was concluded that Bi^3+^ ions prefer to occupy Zn(2) sites. Thus the distance between Bi^3+^ and Eu^3+^ ions can be adjusted through their total concentrations. When their total molar concentration is larger than one sixth of that of Zn sites, Bi^3+^ and Eu^3+^ will locate in two adjacent Zn(2) sites and therefore extreme efficient energy transfer occurred^12^.

However, there are defects because of charge imbalance in Bi^3+^, Eu^3+^ co-doped ZnMoO_4_ phosphor. Additionally, concentration quenching occurred when the total concentration of Bi^3+^ and Eu^3+^ exceed one sixth of Zn sites. To solve the problem of unbalance charge as well as to explore the reasons for luminescence quenching, ZnMoO_4_:Bi^3+^, Eu^3+^, M^+^ (M = Li, Na, K) phosphors were prepared and their properties were investigated. We found that only Na^+^ can strongly enhance the luminescence intensity of Eu^3+^. Based on the previous work as well as the preferred coordination number of Li, Na and K, site occupation of Bi^3+^ and Eu^3+^ was further adjusted and controlled. Thus site occupation preference of Bi^3+^ and Eu^3+^ and energy transfer between them in ZnMoO_4_ were discussed in detail. Moreover, the red emission of Eu^3+^ was further increased after co-doping with charge compensator Na^+^ ions.

## Results

### Crystal structure

**Figure 1:**
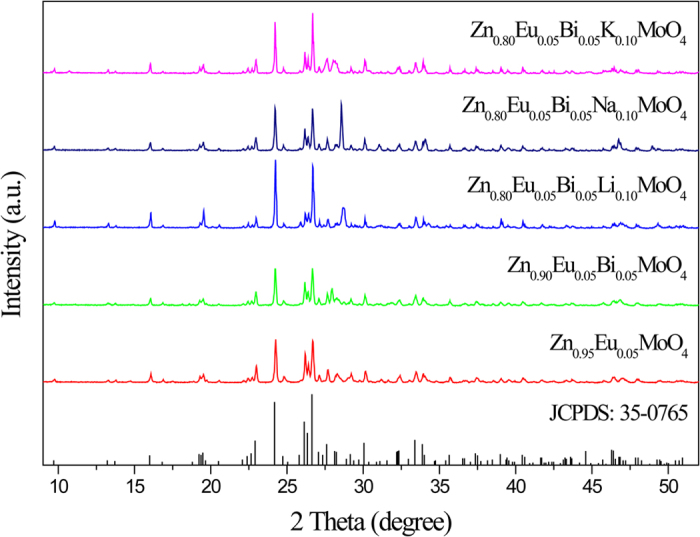
XRD patterns of the ZnMoO_4_, Zn_0.95_Eu_0.05_MoO_4_, Zn_0.90_Eu_0.05_Bi_0.05_MoO_4_ and Zn_0.80_Eu_0.05_Bi_0.05_M_0.10_MoO_4_ (M = Li^+^, Na^+^, K^+^) phosphors were sintered at 700 °C for 3 h.

Figure 1 showed the results of the X-ray diffraction of the product after calcination at 700 °C for 3 h. All the diffraction peaks could be indexed to the triclinic wolframite ZnMoO_4_ phase (JCPDS 35–0765) with space group P-1 and there was no formation of impurity phases. This indicated that the obtained samples were single-phased and the co-doped Eu^3+^, Bi^3+^ and alkali metal ions did not lead to any significant change in the host structure.

**Figure 2:**
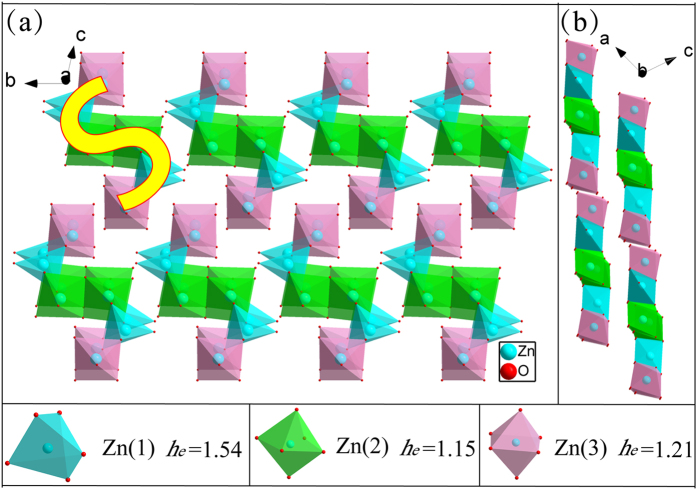
Information of S-shaped clusters in ZnMoO_4_ crystal structure. (**a**) perspective view and (**b**) side view.

As can be seen from Fig. 2, every six Zn-O polyhedra form an S-shaped cluster. The completely centro-symmetric S-shaped cluster has three kinds of Zn sites. Each Zn(1) combine with five O ions to form Zn(1)O_5_ hexahedral. In the center of S-shaped clusters, each two Zn(2)O_6_ polyhedron connected by sharing edges and their relatively distance is only 3.2202 Å. Zn(2) and Zn(3) ions have six coordination number in an approximately octahedral coordination environment. In our previous work, the host lattice environment of luminescence centres strongly affects the luminescence properties. Covalence of chemical bond, coordination number of central ions and site symmetry appear to be important factors for the luminescence properties of Bi^3+^ ion^15^. The environment factor of three kinds of Zn sites were marked in corresponding position in the diagram. The five coordinated Zn(1) site has largest environmental factor value. The environment factor of Zn(2) and Zn(3) sites are very similar with only 5% difference. However, only Na^+^ ions can form five-coordinate structure. Therefore, Bi^3+^, Eu^3+^, Li^+^ and K^+^ ions preferentially occupy Zn(2) sites while only Na^+^ can lie in Zn(1) sites.

### The valence state of bismuth in doped samples

**Figure 3:**
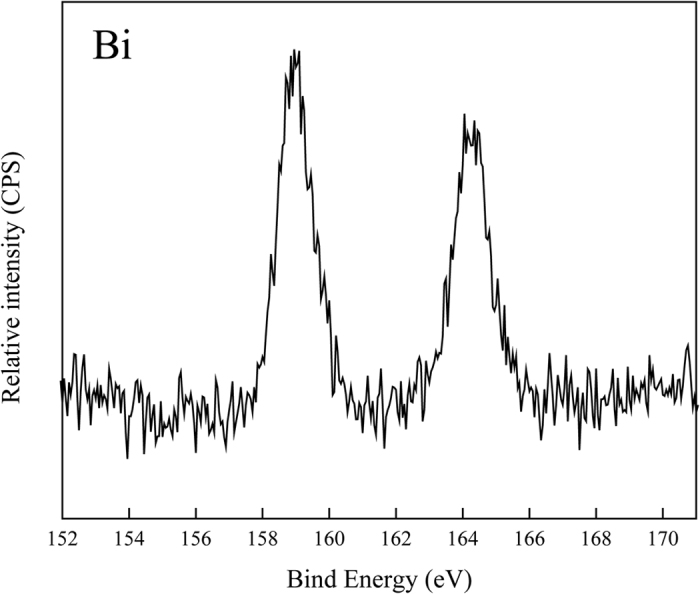
XPS spectra of Zn_0.80_Eu_0.05_Bi_0.05_Na_0.10_MoO_4_ phosphor.

When Bi was introduced into phosphors via higher temperature solid state reaction method, it could readily transform into different valence states. In order to ensure the accuracy of the experiments, it is highly essential to identify the valence of bismuth in the samples. Therefore, we examined a representative sample by XPS. The selected spectrum is shown in Fig. 3, which correspond to Zn_0.80_Eu_0.05_Bi_0.05_Na_0.10_MoO_4_ sample. The XPS of which shows two characteristic Bi^3+^ peaks at around 159.0 and 164.3 eV due to ^4^f_7/2_ and ^4^f_5/2_, respectively. It match well with those of α-Bi_2_O_3_^16^,^17^. It means the dominance of Bi^3+^ in Bi doped ZnMoO_4_ samples.

### Photoluminescence properties

**Figure 4:**
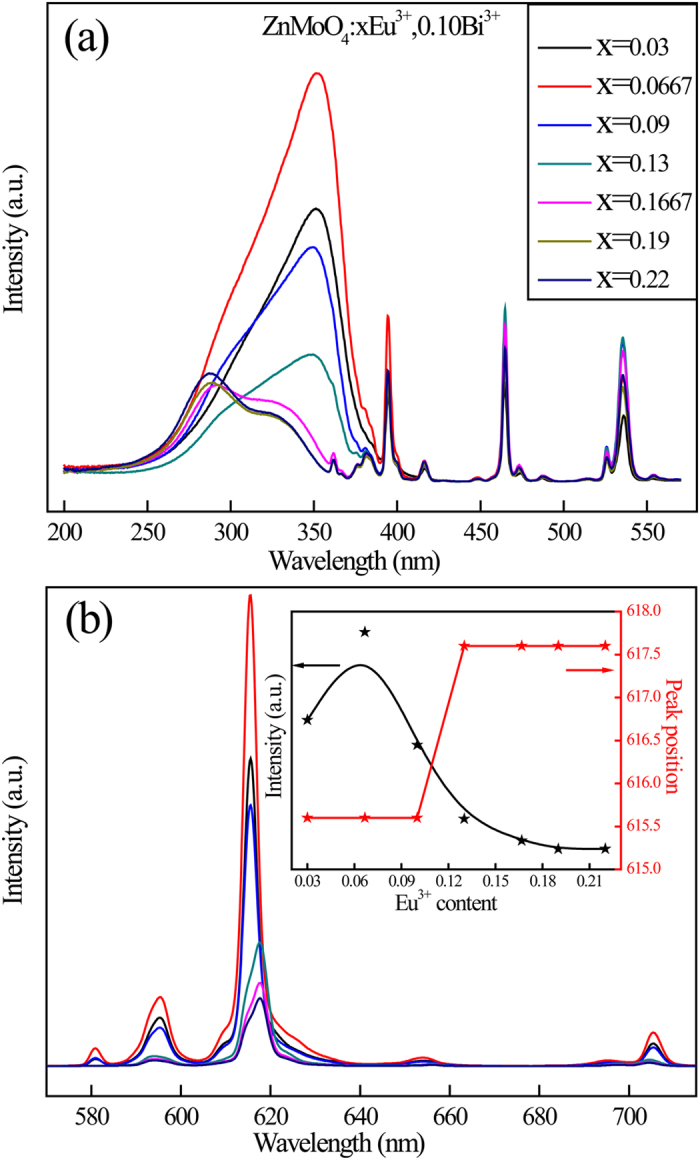
PLE (**a**) and PL (**b**) spectra of Zn_0.90−x_Eu_x_Bi_0.10_MoO_4_ (x = 0.03–0.22) phosphors at room temperature.

In order to investigate the relationship between doping concentrations and luminescence properties, the variation of emission intensity in Zn_0.90−y_Eu_y_Bi_0.10_MoO_4_ phosphors with the increasing Eu^3+^ concentration needs to study. Figure 4(a) showed the excitation spectra of the phosphors monitored at the ^5^D_0_→^7^F_2_ transition emission (616–619 nm) of Eu^3+^. In the PLE spectra, the broad band within the range 250–340 nm could be ascribed to the O^2−^→Mo^6+^ and O^2−^→Eu^3+^ charge transfer band (CTB) transition. The sharp lines were due to the intra configurational 4f-4f transitions of Eu^3+^ ions, which could be assigned to ^7^F_0_-^5^D_4_ (363 nm), ^7^F_0_-^5^L_7_ (385 nm), ^7^F_0_-^5^L_6_ (395 nm), ^7^F_0_-^5^D_3_ (417 nm), ^7^F_0_-^5^D_2_ (466 nm) and ^7^F_0_-^5^D_1_ (537 nm), respectively. In addition, a strong excitation band peaking at about 354 nm in Zn_0.80_Eu_0.10_Bi_0.10_MoO_4_ phosphor was observed due to the ^1^S_0_→^3^P_1_ transition of Bi^3+^. When the total molar concentration was beyond 1/6, Bi^3+^ and Eu^3+^ began to sit two adjacent Zn(2) sites. According to Fig. 4(a), when the Eu^3+^ content was more than 0.0667, the Bi^3+^ ions ^1^S_0_-^3^P_1_ excitation band peaking at about 331 nm decreased suddenly and the peak position of excitation band shifted from 355 nm to 331 nm obviously. The optimal doping concentration of Eu^3+^ was 0.0667. Moreover, the new super energy transfer from Bi^3+^ to Eu^3+^ emerged due to their short distance. According to the inset in Fig. 4(b), the PL intensity increased with increasing Eu^3+^ concentration within the range from 0.03 to 0.0667. However, when the doping concentration of Eu^3+^ was beyond 0.0667, the emission intensity decreased with increasing Eu^3+^ concentration. The concentration quenching occurred due to the short distance between Eu^3+^ ions. This indicated that the over high concentration of Eu^3+^ can result in Eu^3+^ ions occupy two adjacent Zn(2) sites in S-shaped cluster structure.

**Figure 5:**
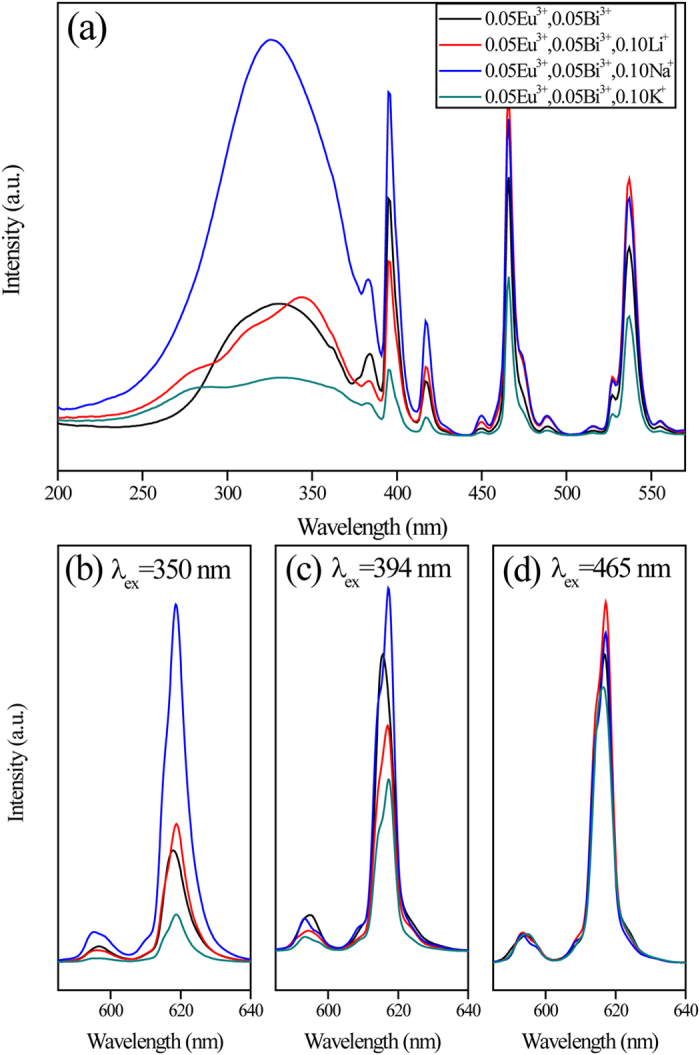
Excitation (a) and emission (b,c,d) spectra of Zn_0.90_Eu_0.05_Bi_0.05_MoO_4_ and Zn_0.80_Eu_0.05_Bi_0.05_M_0.10_MoO_4_ (M = Li^+^, Na^+^ and K^+^) phosphors.

It is widely reported in the literature that the co-doping of alkali ions in the host lattice can enhance the luminescence significantly due to the strongly affects on the crystal structure. In this paper, Li^+^, Na^+^ and K^+^ ions were added to act as the remote control device to adjust the position and crystal structure environment of Bi^3+^ and Eu^3+^ ions in Zn_0.90_Eu_0.05_Bi_0.05_MoO_4_ phosphors. Because alkali metal ions can modify the local symmetry and the surroundings near the rare earth ions, which can significantly affect the photoluminescence properties. The PL and PLE spectra of Zn_0.90_Eu_0.05_Bi_0.05_MoO_4_, Zn_0.80_Eu_0.05_Bi_0.05_Li_0.10_MoO_4_, Zn_0.80_Eu_0.05_Bi_0.05_Na_0.10_MoO_4_, and Zn_0.80_Eu_0.05_Bi_0.05_K_0.10_MoO_4_ phosphors at room temperature are shown in Fig. 5. The photoluminescence excitation (PLE) spectra monitored at the ^5^D_0_-^7^F_2_ transition emission (616–619 nm) of Eu^3+^ were shown in Fig. 5(a). From Fig. 5(a), the excitation spectra of samples are obviously affected by doping ions. Especially, the introduction of Na^+^ ions remarkably enhanced the excitation band intensity in the ultraviolet and near ultraviolet region. According to Fig. 5(b,c,d), the shape and positions were similar in the PL spectra for all samples. When excited at about 350 nm, the PL intensity of Zn_0.80_Eu_0.05_Bi_0.05_Na_0.10_MoO_4_ phosphor enhanced three times compared to the Zn_0.90_Eu_0.05_Bi_0.05_MoO_4_ phosphor. The order of emission intensity for the Eu^3+^ ions with the three alkali metal ions is Na≫Li>K. However, there is no contribution on luminescence enhancement for Li^+^ ions. Furthermore, the introduction of K^+^ can significantly reduce the emission intensity of Eu^3+^. This reflects the impact of the alkali metal ions as remote control device on the energy transfer from Bi to Eu. From Fig. 5(c), when excited at about 394 nm to excite the inner 4f electron transition, the emission spectrum simultaneously affected by energy transfer from Bi to Eu ions due to the extending of Bi^3+^ ions absorption band. The order of emission intensity for the Eu^3+^ ions with the three alkali metal ions is Na≫Li>K.

Without the influence of Bi^3+^ excitation band, the emission spectra which were excited at about 465nm reflect the influence of alkali metal ions as charge compensators on the luminescent center of Eu^3+^ ions. From Fig. 5(d) it can be seen that the influence of doping ions on the fluorescence intensity was different from Fig. 5(b,c). The introduction of Li^+^, Na^+^ and K^+^ ions influenced the luminous intensity of Eu^3+^ ions obviously. The order of emission intensity for the Eu^3+^ ions with the three alkali metal ions is Li>Na>K. This reflects the effect of introduce alkali metal ions as charge compensators. However, from Fig. 5(a,b), only Na^+^ ions can strongly enhance the emission intensity which was different with the effect of charge compensation. Li^+^ and K^+^ ions have hindered the energy transfer from Bi to Eu ions. This shows that there is a special enhancement mechanism different with charge compensation. For further investigating the enhancement mechanism, the doping ions were fixed at Na^+^ ions.

**Figure 6:**
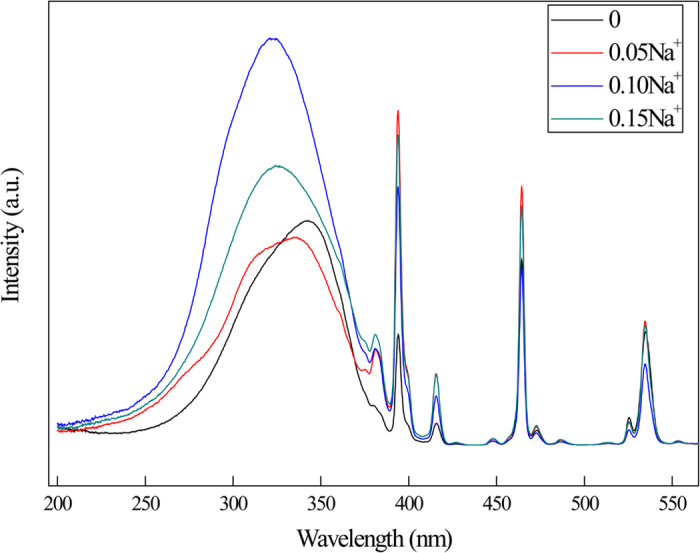
PLE spectra of Zn_0.80−x_Eu_0.05_Bi_0.05_Na_x_MoO_4_ phosphors with various concentration of Na^+^ ions.

**Figure 7:**
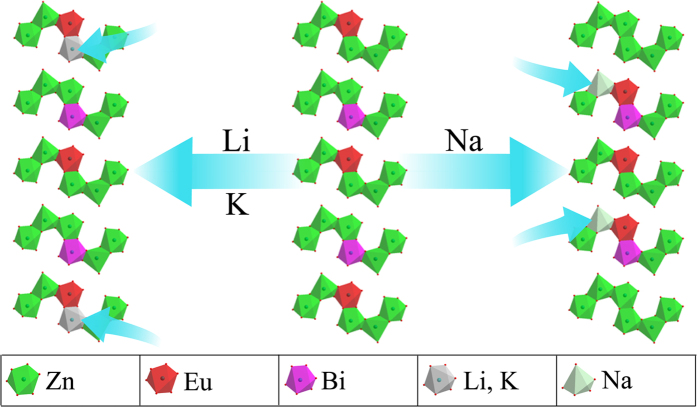
The schematic diagram of luminescence enhancement mechanisms with remote control device.

The excitation spectra of Zn_0.80−x_Eu_0.05_Bi_0.05_Na_x_MoO_4_ phosphors under the excitation wavelength of 614 nm are shown in Fig. 6. From the picture, it can be seen that the introduction of Na^+^ into the host can significantly enhance the luminescence intensity. Therefore, to maintain the charge balance with Na^+^ ions will be more advantageous to improve the energy transfer. However, it was found that the position of excitation band blue-shifted clearly, which indicates that the lattice environment of Bi^3+^ ions has been changed obviously. This is affected by the charge compensation agent. Figure 7 gave the most probable remote control mechanisms.

### Remote control mechanism

The introduction of alkali metal ions can not only lower the charge imbalance but also influence the position of Eu^3+^ and Bi^3+^ ions. According to the previous work, Bi^3+^ and Eu^3+^ ions could occupy preferentially the Zn(2) site^12^. However, because of the charge imbalance when Bi^3+^ or Eu^3+^ seated in the Zn(2) site, alkaline ions will occupy Zn(1) or Zn(2) site which is the nearest dopant neighbor. As reported in literatures, Zn(2) and Zn(3) have six oxygen ligands, while Zn(1) has only five coordination number^18^,^19^,^20^ which can provide more compact space to charge compensation with small ionic radius. Moreover, compared with Zn(2) and Zn(3) site, Zn(1) site has the largest environment factor *h*_*e*_^12^. As far as we know, in alkaline ions (Li^+^, Na^+^ and K^+^), only Na^+^ ions can form five-coordinated polyhedra^21^. Therefore, Li^+^ and K^+^ ions could only occupy Zn(2) site which is nearest Eu^3+^ or Bi^3+^ ions. It is not conductive to shorten the relatively distance between Eu^3+^ and Bi^3+^ ions. Thence, the introduction of Li^+^ and K^+^ can obstruct the energy transfer from Bi^3+^ to Eu^3+^ and then reduce the emission intensity of Eu^3+^ ions. Table 1 lists the ionic radius under different coordination numbers. It can be seen that only Na^+^ ions can form five coordination sites. When Na^+^ ions were added as charge compensators, Zn(1) site was more suitable to be occupied due to the similar ionic radius between Na^+^ (1.14 Å) and Zn^2+^ (0.82 Å) for five coordination. In summary, when Na^+^ was co-doped as charge compensator, it can seat into Zn(1) site and shorten the relative distance between Bi^3+^ and Eu^3+^. The possible mechanism was shown in schematic diagram in Fig. 7.

## Conclusion

In summary, a series of Eu^3+^, Bi^3+^ and alkali metal ions co-doped ZnMoO_4_ phosphors were prepared by the solid-state method in air atmosphere. Li^+^, Na^+^ and K^+^ ions acted as the remote control device were added to improve the luminescence intensities. The super energy transfer process from Bi^3+^ to Eu^3+^ cannot appear only when the total concentration of Bi^3+^ and Eu^3+^ ion is very high. Without alkali metal ions, Eu^3+^ and Bi^3+^ ions lie in Zn(2) sites. The introduced Li^+^ and K^+^ ions seat into Zn(2) site which against the energy transfer from Bi^3+^ to Eu^3+^. However, Na^+^ ions lie in Zn(1) site and then help Bi^3+^ and Eu^3+^ ions to seat into the adjacent Zn(2) sites. Compared with Li^+^ and K^+^ ions occupied Zn(2) sites, Na^+^ ions which preferentially occupied Zn(1) site can get closer to the distance between Bi^3+^ and Eu^3+^ ions. The results indicated that Na^+^ ions provided intensity of energy transfer from Bi^3+^ to Eu^3+^. This mechanism provides a mode how to use remote control device to enhance the energy transfer efficiency which expected to be used to design efficient luminescent materials.

## Experiment

### Materials and Synthesis

A series of ZnMoO_4_:1/6Eu^3+^, 1/6Bi^3+^, xM^+^ (M = Li, Na, K) and ZnMoO_4_:0.05Eu^3+^, 0.05Bi^3+^, 0.1M^+^ (M = Li, Na, K) phosphors with various concentrations and alkali metal ions were synthesized through the solid state reaction method in air atmosphere. The raw materials were ZnO (99%), Eu_2_O_3_ (99.99%), Bi_2_O_3_ (99.9%), Li_2_CO_3_ (99%), Na_2_CO_3_ (99%), K_2_CO_3_ (99%) and MoO_3_ (99%). The starting materials were weighed according to the stoichiometric ratio and well mixed in agate mortar. The mixtures were put into alumina crucible and calcined in muffle furnace at 700 ^o^C for 3 h, and then the white powder phosphor was obtained. All samples were ground into a powder with an agate mortar and pestle for further analysis.

### Materials Characterization

The crystal structure of the samples was determined by the Bruker D8 Advance X-ray diffractometer (Cu Kα_1_ radiation, λ = 0.15406 nm) with radiation at a 0.02^o^ (2θ) /0.05 s scanning step. The photoluminescence excitation (PLE) and emission (PL) spectra were recorded with a Hitachi F-4600 spectrophotometer equipped with a 150 W xenon lamp as an excitation source. All the measurements were performed at room temperature.

## Additional Information

**How to cite this article**: Ran, W. *et al.* Remote Control Effect of Li^+^, Na^+^, K^+^ Ions on the Super Energy Transfer Process in ZnMoO_4_:Eu^3+^, Bi^3+^ Phosphors. *Sci. Rep.* **6**, 27657; doi: 10.1038/srep27657 (2016).

## Figures and Tables

**Table 1:** The ionic radius list with different coordination number^21^.

Ion type	Ionic Radius (Five coordination)	Ionic Radius (Six coordination)
Zn^2+^	0.82 Å	0.88 Å
Mo^6+^	0.64 Å	0.73 Å
Eu^3+^	*	1.087 Å
Bi^3+^	1.1 Å	1.17 Å
Li^+^	*	0.9 Å
Na^+^	1.14 Å	1.16 Å
K^+^	*	1.52 Å
